# Age of sexual initiation and depression in adolescents: Data from the 1993 Pelotas (Brazil) Birth Cohort

**DOI:** 10.1016/j.jad.2017.06.033

**Published:** 2017-10-15

**Authors:** Helen Gonçalves, Ana L. Gonçalves Soares, Isabel O. Bierhals, Adriana K.F. Machado, Mayra P. Fernandes, Roberta Hirschmann, Thais M. da Silva, Fernando C. Wehrmeister, Ana M.B. Menezes

**Affiliations:** a Postgraduate Program in Epidemiology, Federal University of Pelotas, Brazil; b MRC Integrative Epidemiology Unit at the University of Bristol, School of Social & Community Medicine, University of Bristol, Bristol, UK

**Keywords:** Adolescent, Cohort studies, Coitus, Depressive disorder, Sexual and reproductive health, 1993 Pelotas Cohort

## Abstract

**Background:**

Studies have shown that sexual initiation at earlier ages increases the risk of depressive symptoms in adolescents. However, little is known about its association with major depressive episode (MDE).

**Methods:**

The association between age of sexual initiation and MDE at 18 years was assessed in the 1993 Pelotas Birth Cohort using multiple logistic regression. Sexual initiation characteristics (age and type of partner) were assessed at the 15- and 18-years follow-up. The age of sexual initiation was evaluated in categories (11–14, 15–16, 17+ years). The type of partner was categorized into: boyfriend/ girlfriend, casual partner and other. MDE was assessed using the Mini International Neuropsychiatric Interview (MINI).

**Results:**

From the 4027 adolescents assessed, the prevalence of MDE was higher in females (10.1%) than in males (3.4%), and 66.7% of the males and 58.6% of the females reported sexual initiation up to 16 years (p < 0.001). Female adolescents who had sexual initiation <17 years had higher odds of MDE (15–16 years: OR 2.29; 11–14 years: OR 2.23), however no association was found for males. The type of partner in the first sexual intercourse was not associated to depression.

**Limitations:**

Possibility of recall bias on the age of sexual initiation, and low statistical power for some analyses.

**Conclusions:**

A positive association between age of sexual initiation and MDE was observed only in females. More investigation is needed to understand the mechanisms through which age of sexual initiation can affect the risk of depression and whether the association persists in adulthood.

## Highlights

• The prevalence of depression was higher in females (10.1%) than in males (3.4%).
• The association between age of sexual initiation and depression differed by sex.
• Females who had sexual initiation < 17 years had higher odds of depression.
• The type of partner in the sexual debut was not associated to depression.

## 1 Introduction

Physical, psychological and biological changes that occur during adolescence generally arouse questions, and stimulate concerns and behavioral changes regarding relationships and family (World Health Organization, 2016a). In this period of life other events, such as experimentations and challenges, as well as fears and instabilities arising from the affective-sexual relationships (among other contextual and family-related situations) can make adolescents more vulnerable to develop mental disorders (Assis et al., 2014). These disorders affect about 10–20% of children and adolescents in the world, and are the leading cause of disability among young people (World Health Organization, 2016c). When untreated, mental disorders can affect cognitive development and school performance (Millings et al., 2012), apart from other psychosocial consequences.

Depression is a multifactorial cause disorder (including genetic vulnerability and environmental risk factors), which is characterized by symptoms such as sadness, difficulty concentrating, loss of interest in activities that used to be carried out with satisfaction, fatigue, guilt, low self-esteem, and sleep or appetite changes (World Health Organization, 2016d). In 1990 and 2000 depressive disorders were a leading cause of global burden of disease (GBD) and the second cause of years lived with disability (YLDs), accounting for 8.2% (5.9% − 10.8%) of global YLDs in 2010 (Ferrari et al., 2013). Currently, depression is the leading cause of disability worldwide and it is still a major contributor to the overall GBD (World Health Organization, 2016b).

Associations between risk behaviors and depressive symptoms or depression have been largely reported in both males and females from different ages (Bromet et al., 2011, Lehrer et al., 2006, Madkour et al., 2010). Among these behaviors, early sexual initiation is a factor which can lead to other negative outcomes, such as suicidal ideation, sexually transmitted diseases (STDs), pregnancy, and adherence to other risk behaviors, like smoking and excessive consumption of alcohol (Hallfors et al., 2004, Jamieson and Wade, 2011, Mota et al., 2010, Rector et al., 2003, Savioja et al., 2015, Sussman, 2005).

Studies have shown that sexual initiation increases the prevalence of depressive symptoms in adolescents (Hallfors et al., 2005, Jamieson and Wade, 2011, Meier, 2007, Sabia, 2006, Spriggs and Halpern, 2008). The risk is higher in females (Hallfors et al., 2005, Jamieson and Wade, 2011, Meier, 2007, Sabia, 2006, Savioja et al., 2015, Spriggs and Halpern, 2008), as they are usually more influenced by sociocultural norms concerning sexual behavior, and having sex is frequently considered ‘inappropriate’ for girls but not for boys (Borges and Schor, 2005, Sanchez et al., 2012). In addition, mental changes can be influenced by low self-esteem, which is also more common among females (Kiviruusu et al., 2016)

Furthermore, the association of sexual initiation with adolescent's mental health seems to be affected by the type of relationship with the sexual partner (Meier, 2007, Mendle et al., 2013). If the relationship is serious and continues for a while after sexual initiation, it can be positive regarding mental health (Meier, 2007). However, having sex with a partner whose relationship does not involve commitment and/or is of short duration may affect negatively mental health (Meier, 2007, Mendle et al., 2013).

Most of the evidence available on the association between sexual initiation and depression comes from studies carried out in North-America (Jamieson and Wade, 2011, Mendle et al., 2013, Sabia, 2006, Vasilenko et al., 2016). In this sense, it is necessary to assess the association between age of sexual initiation and mental disorders in adolescents in different contexts, as both sexual initiation and mental health problems are influenced by socio-demographic and cultural factors. Thus, the aim of this study was to assess the association between age of sexual initiation and major depression in adolescents aged 18 years from a birth cohort in a middle-income country.

## 2 Methods

### 2.1 The sample

The 1993 Pelotas Birth Cohort recruited all children born alive in hospitals in the urban area of the city of Pelotas, Southern Brazil, between 1st January and 31st December 1993 (Victora et al., 2008). In that year 5265 births were recorded, and 5249 mothers consented to their children to take part in the study. Since the perinatal visit, subsamples of the cohort have been evaluated, and at 11 years the first attempt to interview the full cohort was made. At the 11-year follow-up 4452 adolescents were interviewed (87.5% of the original cohort), at the 15-year follow-up 4325 were assessed (85.7% follow-up rate), and at 18 years 4106 answered the questionnaire (81.3% follow-up rate) (Gonçalves et al., 2014). At the 11-year follow-up the interviews were carried out in the households, and at the 15- and 18-year follow-up the adolescents were interviewed at the headquarters of the study. The questionnaires used for the 1993 Pelotas Birth Cohort are available at <http://www.epidemio-ufpel.org.br/site/content/coorte_1993/>. More detailed information on the methodology applied and the cohort follow-ups are described in other publications (Gonçalves et al., 2014, Victora et al., 2008).

For this study, only people with complete data on age of sexual initiation and depression were included (N = 4031).

### 2.2 Measurements

Major depressive episode (MDE) was measured at the 18-year follow-up using the Brazilian version (version 5.0.0) of the Mini International Neuropsychiatric Interview (MINI), a diagnostic interview instrument. The questionnaire has a recall period of 6 months, was validated for the Brazilian population, and has 0.92 of both sensitivity and specificity (Amorim, 2000, de Azevedo Marques and Zuardi, 2008). The MINI was administered fully structured for trained psychologists who were blind to the exposure status.

Data on sexual initiation characteristics (age of sexual initiation and who was the first sexual partner) was obtained through an anonymized self-completed questionnaire at the 15- and 18-year follow-ups. To assess sexual intercourse, the following question was used: “have you ever had sexual intercourse?”, and sexual intercourse was defined as penile penetration of the vagina or anus. If the adolescent answered affirmatively, it was then asked: “how old were you at your first sexual intercourse?” and “who was your first sexual intercourse with?”, and the response options were: boyfriend/ girlfriend, sex worker, any relative, casual partner (a more open relationship, with less commitment than a boyfriend/girlfriend), housekeeper or another person. The responses were then categorized into: boyfriend/ girlfriend, casual partner and other. Information on age of sexual initiation was used from the 18-year follow-up in order to diminish exaggerated responses and to provide a more accurate information on the age of the first sexual initiation. When the data was missing at 18 years, it was obtained from the 15-year questionnaire (n = 31). Information on who was the first sexual partner was obtained at the 15-year follow-up from those who reported having had sexual initiation.

### 2.3 Covariates

Family income of the month prior to the delivery (quintiles), maternal schooling (0–4 years; 5–8 years; 9–11 years; 12+ years), and maternal age (years) were assessed at the perinatal visit. Quintiles of family income were generated based on the full original sample. Skin color (white, black, and mixed), alcohol experimentation (yes/no), smoking experimentation (yes/no), and maternal mental health were assessed at the 11-year follow-up visit. Maternal mental health was measured using the short-version of the Self-Reporting Questionnaire (SRQ-20), and it was divided into two groups: no common mental disorders (a score less than or equal to seven) and common mental disorders (a score equal to or greater than eight) (Mari and Williams, 1986). Pubertal development was assessed at 15 years using Tanner stages (Tanner, 1966) through an anonymized self-completed questionnaire. Age of menarche was assessed at the 18-year follow-up.

### 2.4 Statistical analysis

Initially, the distribution of the covariates was described, and the prevalence of depression was assessed according to them. Heterogeneity chi-squared test was used to evaluate the difference among the categories, and linear trend was used when appropriate. Logistic regression was used to calculate crude and adjusted odds ratios (OR) and their respective 95% confidence interval (95% CI) for the association between sexual initiation characteristics and depression, and Wald test was used to estimate the significance. We *a priori* decided to stratify the analysis by sex. The analysis was adjusted for all the covariates defined above; age of menarche was used only for analysis carried out for females. The significance level adopted was 5%.

The analyses were performed in the software Stata 14.1® (Statcorp, College Station, TX, USA).

### 2.5 Ethical approval

The 18-year follow-up of the 1993 Pelotas Cohort was approved by the Research Ethics Committee of the Medical School of Federal University of Pelotas under protocol 40600026. After agreeing to take part in the study, the adolescents provided written informed consent.

## 3 Results

**Fig. 1:**
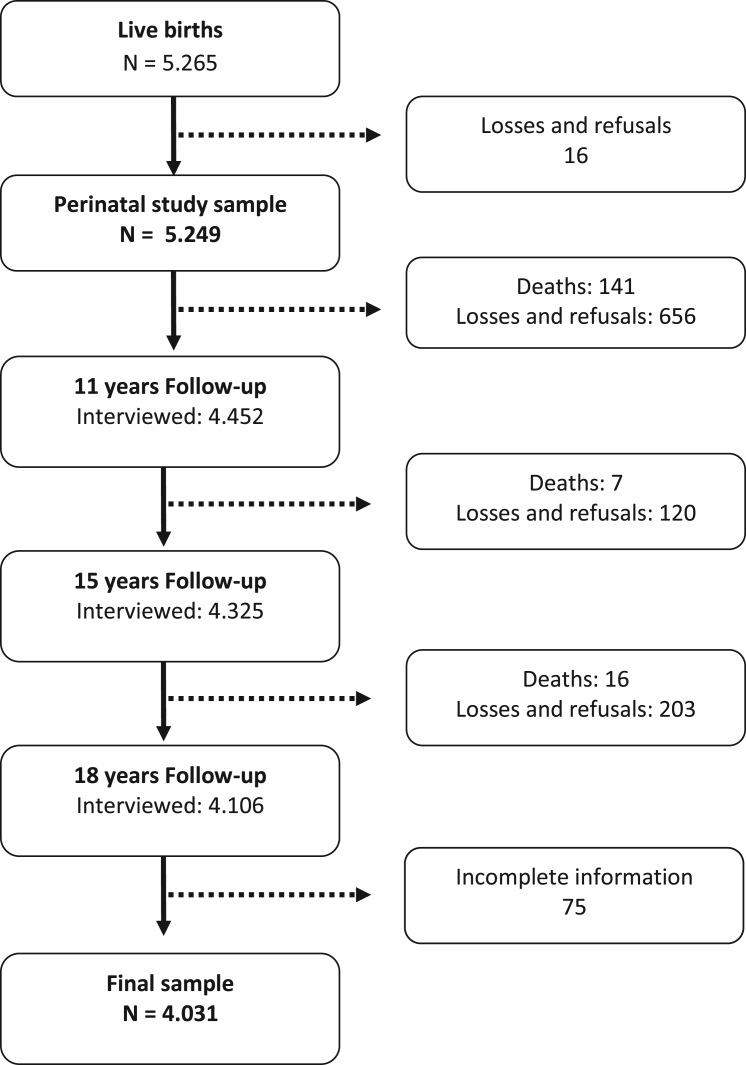
Description of the 1993 Pelotas Birth Cohort and the participants included in this study. 1993 Pelotas Cohort, 1993–2011.

Fig. 1 shows the description of the study population at each follow-up of the cohort. Those excluded from the analysis due to missing data or loss of follow-up (Table 1) were more likely to be male, to belong to the extreme groups (lowest and highest) of family income and maternal schooling (however, the mean family income and mean years of schooling did not differ), and to have higher ages of sexual initiation.

**Table 1:** Socioeconomic and demographic characteristics of the participants included and not included in the analysis due to missing data or loss to follow-up. 1993 Pelotas Cohort, 1993–2011.

**Variables**	**Included in the analysis** **N (%)**	**Not included in the analysis** **N (%)**	**P-value**	**Original sample** **N (%)**
**Sex**			0.018	
Male	1965 (48.8)	641 (52.6)		2606 (49.6)
Female	2066 (51.2)	577 (47.4)		2643 (50.4)
**Family income**			0.038	
1° quintile (lowest)	761 (19.2)	269 (22.9)		1030 (20.1)
2°	928 (23.4)	267 (22.7)		1195 (23.3)
3°	697 (17.6)	192 (16.3)		889 (17.3)
4°	795 (20.1)	207 (17.6)		1002 (19.5)
5° quintile (highest)	781 (19.7)	240 (20.4)		1021 (19.9)
Mean (SD)^a^	4.27 (5.8)	4.40 (5.79)	0.503	4.29 (5.8)
**Maternal schooling (years)**			<0.001	
0–4	1071 (26.6)	396 (32.5)		1467 (28.0)
5–8	1926 (47.9)	499 (41.0)		242 (46.3)
9–11	722 (17.9)	201 (16.5)		923 (17.6)
≥ 12	306 (7.6)	121 (9.9)		427 (8.2)
Mean (SD)	6.8 (3.5)	6.6 (3.8)	0.269	6.7 (3.6)
**Maternal age (years)**			0.528	
< 20	688 (17.1)	228 (18.7)		916 (17.5)
20–24	1102 (27.3)	345 (28.3)		1447 (27.6)
25–29	1046 (26.0)	307 (25.2)		1353 (25.8)
30–34	743 (18.4)	213 (17.5)		956 (18.2)
≥ 35	451 (11.2)	125 (10.3)		576 (11.0)
**Skin color**			0.725	
White	2493 (66.6)	34 (66.7)		2527 (66.6)
Black	562 (15.0)	6 (11.8)		568 (15.0)
Mixed	686 (18.3)	11 (21.6)		697 (18.4)
**Maternal common mental disorders**			0.750	
No	2634 (69.3)	279 (68.5)		2913 (69.2)
Yes	1166 (30.7)	128 (31.5)		1294 (30.8)
**Smoking experimentation**			0.661	
No	3103 (82.7)	320 (81.8)		3423 (82.6)
Yes	648 (17.3)	71 (18.2)		719 (17.4)
**Alcohol experimentation**			0.293	
No	3665 (96.5)	379 (95.5)		4044 (96.4)
Yes	133 (3.5)	18 (4.5)		151 (3.6)
**Pubertal development**			0.902	
1 (less advanced)	59 (1.7)	6 (1.8)		65 (1.7)
2	293 (8.2)	30 (9.1)		323 (8.3)
3	1084 (30.2)	101 (30.5)		1185 (30.3)
4	1434 (40.0)	124 (37.5)		1558 (39.8)
5 (more advanced)	716 (20.0)	70 (21.2)		786 (20.1)
**Age at first sexual initiation (years)**			<0.001	
11–14	1067 (26.5)	70 (19.4)		1137 (25.9)
15–16	1454 (36.1)	8 (2.2)		1462 (33.3)
≥ 17	1510 (37.5)	282 (78.3)		1792 (40.8)
**First sexual partner**			0.264	
Boyfriend/girlfriend	450 (61.1)	44 (53.7)		494 (60.3)
Casual partner	230 (31.2)	28 (34.2)		258 (31.5)
Other	57 (7.7)	10 (12.2)		67 (8.2)

SD: standard deviation.

a Family income assessed in minimum wages.

The socioeconomic, demographic and health characteristics of the adolescents are presented in Table 2. More than half were female and reported white skin color. Regarding the mothers, 74.5% studied at least 8 years, 53.3% were aged 20–29 years when the adolescent was born, and 40.0% had common mental disorders. At 11 years, 17.3% and 3.5% of the adolescents had tried smoking and alcohol, respectively. Males reported more advanced stages of pubertal development at age 15 than females (p<0.001). The majority of the adolescents reported having had sexual initiation by the age of 18, and the prevalence was higher in males (82.3%) than females (77.8%). Among males, 66.7% reported sexual initiation up to 16 years, whilst 58.6% of the females reported sexual initiation by that age (p<0.001). Most females reported a boyfriend/girlfriend as their first sexual partner (83.8%), and 58.4% of the males reported a casual partner or other person as their first sexual partner (p<0.001).

**Table 2:** Socioeconomic, demographic and health characteristics of the adolescents. The 1993 Pelotas (Brazil) Birth Cohort, 2011 (N = 4031).

**Variables**	**Total** N = 4031	**Male** N = 1965 (48.8%)	**Female** N = 2066 (51.2%)	**p-value**^a^
	N (%)	N (%)	N (%)	
***Variables assessed at the perinatal visit***				
**Family income**				0.628
1° quintile (lowest)	761 (19.2)	366 (18.8)	395 (19.5)	
2°	928 (23.4)	475 (24.5)	453 (22.4)	
3°	697 (17.6)	338 (17.5)	359 (17.8)	
4°	795 (20.1)	383 (19.8)	412 (20.3)	
5° quintile (highest)	781 (19.7)	375 (19.4)	406 (20.0)	
**Maternal schooling (years)**				0.727
0–4	1071 (26.6)	509 (26.0)	562 (27.2)	
5–8	1926 (47.9)	943 (48.1)	983 (47.7)	
9–11	722 (17.9)	354 (18.0)	368 (17.8)	
≥ 12	306 (7.6)	156 (8.0)	150 (7.3)	
**Maternal age (years)**				0.474
< 20	688 (17.1)	352 (17.9)	336 (16.3)	
20–24	1102 (27.3)	515 (26.2)	587 (28.4)	
25–29	1046 (26.0)	513 (26.1)	533 (25.8)	
30–34	743 (18.4)	361 (18.4)	382 (18.5)	
≥ 35	451 (11.2)	223 (11.4)	228 (11.0)	
***Variables assessed at the 11-year follow-up***				
**Skin color**				0.296
White	2493 (66.6)	1200 (67.4)	1293 (65.9)	
Black	562 (15.0)	272 (15.3)	290 (14.8)	
Mixed	686 (18.4)	308 (17.3)	378 (19.3)	
**Maternal common mental disorders**				0.125
No	2330 (60.0)	1099 (58.8)	1231 (61.2)	
Yes	1550 (40.0)	770 (41.2)	780 (38.8)	
**Smoking experimentation**				0.076
No	3103 (82.7)	1452 (81.6)	1651 (83.8)	
Yes	648 (17.3)	328 (18.4)	320 (16.2)	
**Alcohol experimentation**				0.096
No	3665 (96.5)	1743 (96.0)	1922 (97.0)	
Yes	133 (3.5)	73 (4.0)	60 (3.0)	
***Variables assessed at the 15-year follow-up***				
**Pubertal development**				<0.001
1 (less advanced)	59 (1.6)	7 (0.4)	52 (2.7)	
2	293 (8.2)	72 (4.4)	221 (11.4)	
3	1084 (30.2)	326 (19.8)	758 (39.0)	
4	1434 (40.0)	708 (43.0)	726 (37.4)	
5 (more advanced)	716 (20.0)	532 (32.4)	184 (9.5)	
**First sexual partner**^b^				<0.001
Boyfriend/girlfriend	450 (61.1)	165 (41.6)	285 (83.8)	
Casual partner	230 (31.2)	183 (46.1)	47 (13.8)	
Other	57 (7.7)	49 (12.3)	8 (2.4)	
***Variables assessed at the 18-year follow-up***				
**Ever had sexual intercourse**				<0.001
No	802 (20.0)	345 (17.7)	457 (22.2)	
Yes	3200 (80.0)	1602 (82.3)	1598 (77.8)	
**Age at first sexual initiation (years)**				<0.001
11–14	1067 (26.5)	605 (30.8)	462 (22.4)	
15–16	1454 (36.1)	705 (35.9)	749 (36.2)	
≥ 17	1510 (37.4)	655 (33.3)	855 (41.4)	

a Chi-squared test for the difference between males and females.

b Data available only for those who reported sexual initiation at the 15-year follow-up (N = 737).

The prevalence of MDE (Table 3) was higher in females (10.1%, 95% CI: 8.8; 11.5) than males (3.4%, 95% CI: 2.7; 4.3). Higher prevalence was also observed in those who reported mixed skin color and who belonged to the lowest quintiles of family income, and the lower the maternal schooling, the higher the prevalence of MDE. A higher prevalence of MDE was observed in males whose mothers had maternal mental health problems, and the prevalence of MDE was similar regardless smoking experimentation, alcohol experimentation, and pubertal development. For females, a higher prevalence of MDE was observed in those whose mothers were both younger (<25 years) and older (≥35 years) at delivery. For females, the lower the age of sexual initiation, the higher the prevalence of MDE. Females who reported sexual initiation with a partner other than a boyfriend/girlfriend or a casual partner tended to have a higher prevalence of MDE (25%).

**Table 3:** Prevalence of major depressive episode (MDE) according to socioeconomic, demographic and sexual initiation characteristics. The 1993 Pelotas (Brazil) Birth Cohort, 2011 (N = 4031).

**Variables**	**Male** (3.4%)		**Female** (10.1%)	
	%	**p-value**^a^	%	**p-value**^a^
***Variables assessed at the perinatal visit***				
**Family income (quintiles)**		0.046		0.002
1° quintile (lowest)	4.1		12.7	
2°	4.8		13.5	
3°	4.1		10.0	
4°	1.8		6.6	
5° quintile (highest)	1.9		7.4	
**Maternal schooling (years)**		0.002^b^		<0.001^b^
0–4	5.5		12.1	
5–8	3.1		11.5	
9–11	2.3		6.3	
≥ 12	1.3		2.0	
**Maternal age (years)**		0.069		0.034
< 20	4.8		14.3	
20–24	4.7		10.2	
25–29	2.1		8.1	
30–34	2.2		8.4	
≥ 35	3.1		11.0	
***Variables assessed at the 11-year follow-up***				
**Skin color**		0.202		0.002
White	3.4		8.6	
Black	2.6		10.7	
Mixed	5.2		14.8	
**Maternal common mental disorders**		0.003		0.105
No	2.4		9.2	
Yes	4.9		11.4	
**Smoking experimentation**		0.850		0.602
No	3.4		10.4	
Yes	3.7		9.4	
**Alcohol experimentation**		0.738		0.083
No	3.4		9.8	
Yes	4.1		16.7	
***Variables assessed at the 15-year follow-up***				
**Pubertal development**		0.506		0.129
1 (less advanced)	0.0		13.5	
2	0.0		12.2	
3	4.0		9.8	
4	3.1		8.0	
5 (more advanced)	3.4		13.0	
**First sexual partner**^c^		0.613		0.411
Boyfriend/girlfriend	5.5		12.3	
Casual partner	4.9		17.0	
Other	2.0		25.0	
***Variables assessed at the 18-year follow-up***				
**Age of first sexual initiation (years)**		0.615		<0.001
11–14	4.0		13.9	
15–16	3.0		12.3	
≥ 17	3.4		6.1	

a Chi-squared test for heterogeneity.

b Chi-squared test for linear trend.

c Data available only for those who reported sexual initiation at the 15-year follow-up (N = 737).

The association between sexual initiation characteristics and MDE is presented in Table 4. Females who reported sexual initiation before 17 years had twice the odds of MDE compared to those who initiated at or after that age, and the association was still evident after adjustment for confounders. For those who had the sexual initiation at 15–16 years the OR was 2.29 (95% CI: 1.53; 3.41), and for those who had sexual initiation at 11–14 years the OR was 2.23 (95% CI: 1.43; 3.49). Females who had sexual initiation with a partner other than a boyfriend/girlfriend or casual partner had 3.2 the odds of MDE than those who had sexual initiation with a boyfriend/girlfriend, however the confidence interval was very wide (OR 3.22, 95% CI: 0.30; 35.01). No association of age of sexual initiation or first sexual partner with MDE was observed in males.

**Table 4:** Unadjusted and adjusted association between sexual initiation characteristics and major depressive episode (MDE) in adolescents. The 1993 Pelotas (Brazil) Birth Cohort, 2011 (N = 4031).

	**Male**				**Female**			
	Crude OR (95% CI)	p-value^a^	Adjusted^b^ OR (95% CI)	p-value^a^	Crude OR (95% CI)	p-value^a^	Adjusted^c^ OR (95% CI)	p-value^a^
**Age of first sexual initiation (years)**		0.618		0.473		<0.001		<0.001
11–14	1.18 (0.66; 2.14)		0.92 (0.46; 1.82)		2.48 (1.69; 3.65)		2.23(1.43; 3.49)	
15–16	0.88 (0.48; 1.62)		0.65 (0.31; 1.34)		2.16 (1.52; 3.08)		2.29(1.53; 3.41)	
≥ 17	1		1		1		1	
**First sexual partner**^d^		0.634		0.825		0.429		0.482
Boyfriend/ girlfriend	1		1		1		1	
Casual partner	0.90 (0.35; 2.32)		0.86 (0.26; 2.85)		1.46 (0.63; 3.39)		1.51 (0.54; 4.21)	
Other	0.36 (0.04; 2.92)		0.49 (0.05; 4.70)		2.38 (0.46; 12.26)		3.22 (0.30; 35.01)	

a Wald test derived from logistic regression.

b Adjusted for skin color, family income, maternal age, maternal schooling, maternal common mental disorders, pubertal development, and alcohol and smoking experimentation at 11 years.

c Adjusted for skin color, family income, maternal age, maternal schooling, maternal common mental disorders, pubertal development, alcohol and smoking experimentation at 11 years, and menarche age.

d Data available only for those who reported sexual initiation at the 15-year follow-up (N = 737).

## 4 Discussion

This study showed a positive association between sexual initiation before 17 years and MDE in females but not in males. About 7% of the adolescents had MDE at 18 years, and the prevalence was higher in females (10%). Most of the adolescents had sexual initiation up to 16 years (62.6%), and males reported lower ages of sexual initiation.

Brazilian studies have demonstrated that between 20% and 28% of the adolescents aged up to 17 years reported sexual intercourse, and the prevalence is higher among males than females (Borges et al., 2016; Instituto Brasileiro de Geografia e Estatística (IBGE), 2016). Comparing data from the National School Health Survey (*Pesquisa Nacional de Saúde do Escolar* – *PeNSE*) we observe an increase in the report of sexual intercourse among adolescents aged 13–15 years, from 20.5% in 2009 to 28.7% in 2012 (Malta et al., 2011, Oliveira-Campos et al., 2014). However, in most high-income countries no temporal trend (2002, 2006 and 2010) regarding increase in the prevalence of sexual intercourse at age ≤13 years has been observed (Ramiro et al., 2015). Analysis carried out in six middle-income countries from the Caribbean (Antigua and Barbuda, Dominica, Grenada, Saint Lucia, Saint Vincent and the Grenadines, Trinidad and Tobago) showed that 21.4% of the adolescents from 13 to 16 years reported sexual intercourse (Peltzer and Pengpid, 2015). In the United States, a high-income country, higher prevalence was observed in a study carried out with adolescents aged ≥14 years, which found that 39% reported sexual intercourse (Kaplan et al., 2013). These data show that comparisons should be made cautiously, since the relative timing during adolescence varies by country.

The association observed between age of sexual initiation and depression differed according to sex in the 1993 Birth Cohort, and this was also shown in other longitudinal studies. The National Longitudinal Study of Adolescent Health (Add Health) – a school-based longitudinal study of a nationally representative sample of adolescents in grades 7–12 in the United States – found a positive association between early sexual initiation (usually before 16 years) and depressive symptoms in female adolescents (Hallfors et al., 2005, Kim, 2016, Meier, 2007, Spriggs and Halpern, 2008) and young adults (24 years) (Vasilenko et al., 2016). In the South Korean context, a conservative environment concerning social rules for sexual initiation, a longitudinal study carried out with 3449 adolescents (13–19 y; mean age: 16.2 ± 1.7 years) from the eighth grade found that earlier ages of sexual initiation (before high school) were associated with poorer mental health outcomes (aggressive behavior and depressive symptoms) in female adolescents (Kim, 2016). A Finnish cross-sectional study found that self-reported depression in adolescents aged 14–16 years was associated with having experienced sexual intercourse in both sexes (Savioja et al., 2015). However, some other studies using data from the Add Health found that early sexual initiation was not associated with depressive symptoms in early adulthood (Jamieson and Wade, 2011, Kugler et al., 2015, Spriggs and Halpern, 2008). Therefore, it is possible that this association occurs during adolescence, but does not persist in adulthood, as observed in a longitudinal study (Kugler et al., 2015). Some authors suggest that both early sexual intercourse and depressive symptoms are concomitant outcomes of biopsychosocial processes, and early sexual intercourse could be used as a marker, but not cause of depressive symptoms (Spriggs and Halpern, 2008).

The higher prevalence of MDE observed in females who had sexual intercourse before age 17 could be explained by several reasons. Sexual initiation can occur with partners from different ages and/or social status, which might reduce the possibility of negotiating the postponement of engaging in sexual activity (Borges and Schor, 2005), and this can be even more difficult for females, especially in younger ages. Qualitative studies have found that many female adolescents are unprepared for the first sexual intercourse, and this experience is sometimes unexpected, unwilled, and can occur due to their boyfriends’ insistence (Hawes et al., 2010, Templeton et al., 2016). Moreover, a more conservative social and cultural context can also contribute to the higher risk of depressive symptoms (Kim, 2016), as open talks about sexuality within the family or social groups are usually avoided, uncomfortable or yet not allowed. Despite the growing movement for gender equality, there is still a culturally less favorable position of females towards males about sexuality, which can be noticed also in the different ages of first sexual intercourse between males and females (Crawford and Popp, 2003, Heilborn, 2006). Additionally, emotional symptoms, which are characteristics of depression, can be expressing many of the difficulties of breaking or adapting to the current social rules in the socialization process for both males and females. Therefore, other factors, such as family and social environment (Bromet et al., 2011, Kessler et al., 2001) are important to be investigated in order to better understand the association between age of sexual initiation and depression, since other health risk factors as use of tobacco and drugs, and no use of condoms and contraceptives are also related to early sexual initiation (Borges et al., 2016, Kaplan et al., 2013).

Although several studies have assessed sexual initiation among adolescents, there is no consensus over the definition of early sexual intercourse, and this can be based on the distribution of the age of sexual initiation in each country, the age considered yet emotionally immature, or before the legal age of consent. In general, studies consider ≤14 years (Gonçalves et al., 2015, Kugler et al., 2015) or <16 years as an early age of sexual initiation (Donahue et al., 2013, Spriggs and Halpern, 2008). However, the use of a threshold to define early sexual initiation may not identify some groups which can also have a higher risk of poorer health outcomes, as observed in this and other studies (Kugler et al., 2015, Spriggs and Halpern, 2008, Vasilenko et al., 2016). Using our data to assess the same association between age of sexual initiation and depression, but defining early sexual initiation as ≤14 years, the results are similar for males (OR 1.14, 95% CI 0.62, 2.08), however the risk in females seems to be much smaller (OR 1.37, 95% CI 0.96, 1.96), as a high-risk group for depression (15–16 years) is included in the reference group. It is known that younger individuals tend to have greater difficulty to cope with the responsibilities concerning sexual activity onset and its consequences, as well as with affective relationships and its possible dissolution compared to older individuals or adults (Meier, 2007). Thus, authors should explore different ages or age groups rather than defining sexual initiation as early or not. In the context of sexuality, the social prescriptions that qualifies sexual initiation do not always consider biological and maturation period in this process. Hence, authors should point the importance of taking into consideration sexual scrips, as they inform how adolescents express, identify and judge masculine and feminine sexual behaviors in their context (Templeton et al., 2016).

Our results did not confirm the initial hypothesis that the type of relationship in the first sexual intercourse may influence the risk of depression in females. Other studies show that girls are more likely to have their sexual initiation with a so-called boyfriend, whilst boys more commonly have their first sexual experience with other people, like a friend, a known adult or a sex worker, and also are more often motivated by arousal, curiosity, desire of not being virgin anymore or social status among their peers (Borges and Schor, 2005, 2007; Hawes et al., 2010; Templeton et al., 2016). Some studies have emphasized that girls tend to have sexual intercourse with people whose relationship involves some kind of love commitment, aiming intimacy, closeness, trust and emotional support, reproducing a romantic notion of sex and a kind of sexual “succumbing” to a special person (Borges and Schor, 2005, Hawes et al., 2010, Joyner and Udry, 2000, Templeton et al., 2016, Tolman and McClelland, 2011). Nevertheless, the effects of the type of relationship with a partner on depression can be interpreted and analyzed in a bidirectional way, since both the involvement and the dissolution of the relationship can increase the vulnerability to depressive symptoms by the freshness, immaturity and difficulties in controlling and managing these events by adolescents (Larson et al., 1999). Thus, contextual factors of the first sexual experience and after that can help to understand the association between age of sexual initiation and depression.

Some limitations in our study have to be highlighted. The lack of information on when more severe episodes of depression occurred limited the analysis of a longitudinal association and, thus causal inference. The information about age of sexual intercourse was assessed primarily at the 18-year follow-up, so the possibility of recall bias cannot be discarded. However, sexual initiation is a markable event that is usually remembered with considerable precision, and the information assessed at the 15-year follow-up was avoided due to the possibility of exaggerated answers, especially by males. For those who only had information on age of sexual initiation at the 15-year follow up and have not had sexual intercourse by that time, it was not possible to know when the sexual initiation occurred. However, sensitivity analysis assuming they all had no sexual initiation or had sexual initiation after 17 years showed similar results. The use of self-administered questionnaires to assess sexual behavior can also imply in information bias, such as overestimation on pubertal development and underestimation on the age of sexual initiation in males. We nevertheless believe that the use of an anonymized questionnaire may have reduced this bias, as both male and female adolescents feel more comfortable to answers about this subject compared to a questionnaire applied by interviewers. It has been shown that although self-assessment of sexual maturation has a higher validity in girls, boys overestimate their self-reported sexual maturation compared to physician assessments and hormone levels (Chavarro et al., 2017, Rasmussen et al., 2015). However, self-assessments are considered sufficiently accurate for large epidemiologic studies to distinguish between prepuberty and puberty (Chavarro et al., 2017, Rasmussen et al., 2015). Furthermore, the age at onset of puberty seems not to be related to depression in boys (Wang et al., 2016), thus the association between age of sexual initiation and depression is less likely to be affected by the overestimation of sexual maturation in boys.

In our study, some cases of sexual abuse might have been reported as sexual initiation. However, the prevalence of sexual abuse is low in our cohort (1.5%), and the results do not change when those sexually abused are excluded from the analysis. Even though the cohort has a high follow-up rate, those not included in the analysis were more likely to be male and to have higher age of sexual initiation, however the association found between age of sexual initiation and depression is less likely to have been affected (Nohr et al., 2006). The sample size used for some of the analyses may have limited the power to find associations, especially in relation to the type of first sexual partner, which data was available only for those who reported sexual intercourse at the 15-year follow-up. On the other hand, this study used data from a middle-income country birth cohort with a high follow-up rate, and applied a diagnostic interview instrument to assess MDE. Most of the previous studies have assessed only depressive symptoms (Jamieson and Wade, 2011, Kugler et al., 2015, Meier, 2007), and the one which assessed self-reported lifetime diagnosis of depression did so in young adulthood (Vasilenko et al., 2016). Moreover, all studies were carried out in high-income countries, where the confounding structure for the association between age of sexual initiation and depression can be different.

Even though the mean age of sexual initiation has decreased over the past decades, in younger cohorts it has not changed much (Liu et al., 2015), and recent data show that sexual initiation has occurred in similar ages as observed in this study (Borges et al., 2016, Liu et al., 2015, Malta et al., 2011, Oliveira-Campos et al., 2014). Moreover, although some studies show that the prevalence of depression has increased in adolescents (Mojtabai et al., 2016, Thapar et al., 2012), data from Canadian adolescents did not support a change in MDE prevalence (Wiens et al., 2017). Hence, a cohort effect is less likely to have occurred in the association between age of sexual initiation and depression.

Given the higher odds of MDE in female adolescents who had sexual initiation before 17 years, and considering the increase in sexual intercourse prevalence at earlier ages, it is important to better understand the mechanisms through which the association between age of sexual initiation and depression can occur, and whether the increased risk persists in adulthood in different socio-cultural backgrounds. However, the association between sexual initiation and depression can only be better understood through analyses that explore in detail individual factors related to the reasons why sexual initiation occurred, as well as the family and social environment (such as cultural relevance given to sexual initiation, its age of occurrence, type of relationship with the sexual partner, and family expectations for the adolescent's relationships). What has been socially accepted in terms of behavior for younger ages, which varies for each context in terms of age of sexual initiation, and how this rule is assimilated by girls and boys should be advocated in health and education actions for adolescents.

## Contributors

HG, ALGS, FCW and AMBM designed the study. AKFM, MPF, RH, TMS and ALGS conducted the literature search. ALGS, HG, IOB, MPM and AKF conducted the statistical analysis and interpreted the results. RH, TMS and IOB wrote the first draft of the manuscript. ALGS, HG, FCW and AMBM reviewed the final manuscript and did substantial contributions. All the authors reviewed and approved the final manuscript.

## Role of the funding source

All the founding sources had no role in the study design, collection, analysis or interpretation of the data, writing the manuscript, or the decision to submit the paper for publication.

## Conflict of interest

Nothing to declare.
